# Some Medical Problems in China To-Day

**Published:** 1950-01

**Authors:** Coralie Rendle-Short

**Affiliations:** Obstetrical Specialist, H.M. Colonial Medical Service, Hong Kong


### SOME MEDICAL PROBLEMS IN CHINA TO-DAY

BY

CORALIE RENDLE-SHORT, M.B., CH.M., M.R.C.O.G.
*Obstetrical Specialist, H.M. Colonial Medical Service, Hong Kong.*

---

It is nine o'clock in the evening—as I write I can see the queue starting to form for the out-patient session to-morrow. The men are coughing and spitting, and making themselves as comfortable as possible against the wall, whilst the women squat with undersized infants tied on to their backs. By the morning the crowd will have grown to a great throng waiting for the hundred-odd new tickets that will be given out so that they may consult a doctor. Many come up day after day in vain, others do not want the ticket for themselves—they will wait all night and then sell their place for several dollars to a late comer.

Meanwhile a private practitioner has just been in bemoaning that he has not really got room in his two houses to put the third grand piano he has bought for his children. He is an excellent doctor, and well trained, but has no time to give to the poor struggling masses; he is already more than occupied with his own patients.

Such are the problems of China; the poor so very poor and ignorant, needing and yet suspicious of foreign medicine: the rich very rich, and yet not enough well-trained doctors to treat any section of the community really adequately.

The problems in Hong Kong and in China itself are not, at the moment, quite the same. There are two kinds of doctors allowed to practise in the Colony. First, there are those who have a degree that would be recognized in the United Kingdom, the majority of these being Hong-Kong trained, with or without post-graduate experience elsewhere. There are also Chinese "doctors" or herbalists, who only use traditional Chinese drugs and methods. They are not allowed to use dangerous Western drugs. A great number of patients of all classes try these first, before resorting to foreign ideas. Patients with palpable abdominal tumours often come up with many tiny skin burns made by lighted joss-sticks, or they have sticky black plasters applied to the umbilicus. Those with headache or sore throat have red weals made by special pinching of the skin, often with the idea of letting out a devil.

When Hong Kong was occupied by the Japanese the students in training mostly scattered to universities in Free China, or to India, where they continued their studies and took a Chinese qualification. As many wished eventually to practise in British territory such as Malaya or Hong Kong, they returned to study once more at Hong Kong University. I was assisting in examining about twenty-five of these last June, who were already qualified doctors by Chinese standards. They had had a long and difficult career, many having done excellent work as interpreters or with the Red Cross as well as graduating. On the whole the standard was satisfactory. The best were every bit as good as final-year students in England.

The problem of coping with the sick population or undertaking any preventive medicine is much more difficult. At present the Colony is overcrowded to a degree past belief, probably over a million people living here. These are partly refugees from China, either on account of war or floods; or they simply come because there is comparative safety here, and great prosperity. Fortunes can be made, and work is plentiful. Some charter special four-engined planes to bring them and their families, and these are willing to pay £150,000 to buy a house, or £5,000 key money for a flat; others come by train or boat with their belongings in a big string bag, tied on to a pole. They will occupy a " bed-space " in an already grossly overcrowded room, or put up a hut on any available waste land, so becoming squatters. There are thousands of these dwellings with no water supply or sanitation. Many huts are on roof-tops and all waste is just chucked into the street below, constituting a terrible menace to health. Dysentery, intestinal parasites, skin diseases, and under-nourishment are very common, but the worst problem of all is tuberculosis.

It is most difficult to say how much free or cheap treatment should be given to these people. The more that are treated in the big hospitals, the more come from over the border. It is an unending problem. Apart from the ordinary diseases there are also special cases to be dealt with, such as smallpox, malaria, leprosy or outbreaks of cholera and typhoid fever. Mass free inoculation helps to prevent some of these, and the Government keep the most careful watch on the water supply. It is tested several times daily, so is virtually safe to drink straight from the tap, which for an Eastern city is remarkable.

At present there are two main Government general hospitals, a large fever and mental hospital, and various out-patient clinics. There are also several Chinese hospitals, a tuberculosis sanatorium and some smaller private and mission hospitals run by various religious bodies. There are numerous small nursing and maternity homes for Chinese patients only—often very expensive.

Fully qualified dentists are also in very short supply, and appointments have to be made for three months ahead.

Unfortunately, new doctors coming to the Colony find it almost impossible to get anywhere to live, and have to pay a fabulous amount to get an office. Good reliable drugs from the best houses, both British and American, can usually be purchased, but rubber goods and surgical instruments are about as difficult to get as in England. As well, there are great numbers of strange concoctions such as "Insanity Mixture" and "Vitamins"—"Hormones", which seem to have a tremendous vogue at present and one is obviously expected to prescribe them. The Chinese are great believers in injections, and usually doubt the efficacy of drugs given by mouth.

Now for China itself. The future?—no one knows. There have been certain first-class medical schools which gave an excellent general training; amongst these were the Peking Union Medical College, on American lines, a Canadian school at Tsinan, several medical schools in Shanghai, one in West China, and one in Canton. The P.U.M.C. has recently restarted, and several of the others have made abortive attempts to do so. The latest news from Peking is that the college is still working under the Communists, as they are known to be desperately short of doctors. Difficulties will no doubt arise, both financial—it has been partly supported from America—and in providing good teachers, in maintaining discipline, and in getting good drugs, since foreign trade at the moment is at a standstill. Although good vaccines are made in China, they have to rely a great deal on imported drugs. Now English is being banned as a language for teaching and commercial purposes, so new text books may have to be found. Information given by those in the North is that the medical and nursing courses are in some cases being seriously curtailed to meet the shortage of trained medical personnel.

There used to be also a number of second-class medical schools in smaller provincial towns, many kept up by the Japanese, with lectures in Chinese and badly printed text books, many in German. These continued under the Nationalist regime and gave the poorest possible medical education. Graduate students had never seen an abdomen opened. Their only idea of treating a fracture was to put it in a splint. Doctors from these schools are numerous, and practise in many of the smaller towns, with the results that might be expected.

Whoever governs China will have enormous problems to deal with. The large towns have some well-qualified doctors, but the rural areas could not be worse provided for. The various mission hospitals have done sterling work, but now, although allowed in many cases to carry on, they are much discouraged and have the greatest difficulties to face from the authorities. Organizations such as U.N.R.R.A. and W.H.O. tried to help by sending drugs such as penicillin, also dried milk and other food to particularly needy areas, but their gifts have been much abused, and quantities were sold or given to people for whom they were never intended.

In certain districts such as Northern Honan, special diseases such as kala-azar are very prevalent. The Friends' Ambulance Unit and W.H.O. both sent teams which give vast numbers of injections and other treatment for this, with excellent results. Everywhere tuberculosis and venereal disease are a terrible scourge. Methods of treatment are totally inadequate. The Chinese are a difficult people to educate along public health lines. Noisy clearing of the throat, and spitting are the fashion amongst all classes. The amahs and nurses are often seen discreetly spitting into the sink or baby's bath in the best establishments! Human excretion is always saved for fertilization of the fields. It is dried in open spaces, often near towns, with smells and flies resulting. It is then put back on the plants so that intestinal parasites are universal, and wells become contaminated. After a meal in a restaurant a hot wet towel is passed to every diner, who wipes his sweating face and hands with it, so skin diseases and eye troubles such as trachoma are propagated.

In China anyone is allowed to set up as a doctor whatever type of training he or she has had. Three years ago I did a survey of all the surgeries of any size in a country town of about 100,000 inhabitants in North China. Some were reasonably clean and had hard wooden chairs and benches, and large gruesome anatomical diagrams hung on the walls. Shelves contained rows of bottles and scales and often charms. The best training that any " doctor " had was somewhat equivalent to a theatre porter at home; that is, one or two had worked in a mission hospital and had learned to give anaesthetics and put on splints. None had attended a medical college of any kind, and most had no training whatsoever. Yet they used a mixture of Chinese drugs and whatever foreign ones they could get—particularly penicillin, which they seem to think cures everything.

In the obstetrical and gynaecological world the major problems are sterility and the high infant mortality rate. Prolapse of the uterus is very rare in the North where the women do little manual labour, and much more common in the South where they work very hard on boats, unloading ships, in the fields and on the roads. Carcinoma of the cervix and ovarian cysts are fairly common everywhere. Fibroids are often seen, but not so frequently as in England. Pelvic endrometriosis is rare.

Sterility in China on the part of the woman is ground for divorce; or the husband may, if wealthy, get another wife. The most usual history is that of a woman who had one or two children quite quickly after marriage, they both died, and now years go by and no further pregnancy takes place. On examination the tubes and ovaries and uterus are completely fixed by adhesions, and it is very difficult to know how to help these patients. If any operation is advised, the woman is unable to give her own consent and fathers, mothers-in-law, brothers and sons must all be consulted. In urgent cases, such as a ruptured ectopic, which is quite common, this delay is exasperating; the husband is often quite unable to say anything.

Semi-Westernization such as is seen in big cities, particularly Hong Kong, also makes treatment difficult. Patients in the north and in rural areas all feed their babies at the breast, and are in fact horrified if in certain cases such as open tuberculosis you suggest something else. In Hong Kong women come into hospital armed with their tins of well-known brands of baby foods, and say they have no intention of feeding or looking after their own children—they have amahs to do this for them. The poorer ones take the attitude: " You persuade us to have ante-natal care and come into hospital for confinement—very well, we sit back and do nothing ", and they will not even try to breast-feed. With street hoardings and cinema screens shouting the value of this or that particular brand of dried milk, it is difficult for the foreign doctor in hospital to persuade them otherwise. Consequently outbreaks of infantile diarrhoea and vomiting are always occurring.

One last problem may be mentioned: it is almost impossible to get blood donors amongst the Chinese. Several husbands have bluntly told me, on explaining how serious is the wife's condition: " Well, if she dies I can easily get another ". If a donor is eventually persuaded to give blood, the bottle must be well covered and he must never know how much he gave. I had once finished taking about a pint from a healthy policeman, when he called out: " Be sure you stop when you have taken 5 cc. or my health will be ruined for ever ". Fortunately he never knew the truth, and his health remained good!

Such are some of the medical problems in China—at the moment the answers must in the main lie with the Chinese themselves.

